# Antigenic Diversity of Human Sapoviruses

**DOI:** 10.3201/eid1310.070402

**Published:** 2007-10

**Authors:** Grant S. Hansman, Tomoichiro Oka, Naomi Sakon, Naokazu Takeda

**Affiliations:** * National Institute of Infectious Diseases, Tokyo, Japan; † Osaka Prefectural Institute of Public Health, Osaka, Japan

**Keywords:** Sapovirus, genogroup, genotype, virus-like particles, capsid protein, antiserum, cross-reactivity, research

## Abstract

Sapovirus (SaV) is a causative agent of gastroenteritis. On the basis of capsid protein (VP1) nucleotide sequences, SaV can be divided into 5 genogroups (GI–GV), of which the GI, GII, GIV, and GV strains infect humans. SaV is uncultivable, but expression of recombinant VP1 in insect cells results in formation of viruslike particles (VLPs) that are antigenically similar to native SaV. In this study, we newly expressed SaV GII and GIV VLPs to compare genetic and antigenic relationships among all human SaV genogroups. Hyperimmune antiserum samples against VLPs reacted strongly with homologous VLPs. However, several antiserum samples weakly cross-reacted against heterologous VLPs in an antibody ELISA. Conversely, an antigen ELISA showed that VLPs of SaV in all human genogroups were antigenically distinct. These findings indicate a likely correspondence between SaV antigenicity and VP1 genogrouping and genotyping.

## Abstract 2

Correspondence between virus antigenicity and capsid (VP1) genogrouping and genotyping is likely.

The family *Caliciviridae* contains 4 genera (*Sapovirus*, *Norovirus*, *Lagovirus*, and *Vesivirus*), which include *Sapporo virus*, *Norwalk virus*, *Rabbit hemorrhagic disease virus*, and *Feline calicivirus*, respectively. Sapoviruses (SaVs) and noroviruses (NoVs) are etiologic agents of human gastroenteritis. The prototype strain of human SaV, Sapporo virus, was originally discovered in an outbreak in an orphanage in Sapporo, Japan, in 1977 (*1*). SaV infects children and adults and has been found to cause outbreaks of gastroenteritis in daycare centers, healthcare facilities, and elementary schools. Detection methods include reverse transcription–PCR (RT-PCR), real-time RT-PCR, enzyme immunoassays, and ELISAs (*2*–*6*). Recently, we detected SaV in untreated wastewater samples, treated wastewater samples, and river samples (*7*).

The SaV genomes are predicted to contain either 2 or 3 main open reading frames (ORF1–3). SaV ORF1 encodes for nonstructural proteins and the major capsid protein, and ORF2 (VP2) and ORF3 encode proteins of yet unknown functions. On the basis of VP1 nucleotide sequences, SaVs have been divided into 5 genogroups (GI–GV), of which GI, GII, GIV, and GV strains infect humans and GIII strains infect porcine species (*8*). SaV genogroups can be further subdivided into genotypes. Recently, we identified several recombinant SaV strains (*8*,*9*).

Human SaV and NoV strains are uncultivable, but expression of a recombinant subgenomic-like construct (i.e., VP1 to the end of the genome) or VP1 alone in insect or mammalian cells results in the formation of viruslike particles (VLPs) that are morphologically similar to native SaV (*10*–*16*). However, production of VLPs of SaV remains difficult, usually only resulting in low yields of VLPs compared with norovirus (*10*,*12*,*16*,*17*). Cryoelectron microscopy and x-ray crystallography analyses of NoV VLPs identified the shell (S) and protruding domains (subdomains P1–1, P1–2, and P2) (*18*). Also, Chen et al. described strictly and moderately conserved amino acid residues in the capsid protein among the 4 genera in the family *Caliciviridae* (*13*).

Previously, we reported that SaV GI/1 (strain Mc114) and GV/1 (strain NK24) were antigenically distinct (*5*,*10*). More recently, we discovered that SaV GI/5 (strain Yokote1) VLPs were antigenically distinct from SaV GI/1 Mc114 and GV/1 NK24 VLPs (*19*). Other than these few studies, little is known about the genetic and antigenic relationships among the 4 human SaV genogroups. For classification of NoV, distinct genotypes have been defined as having bootstrap values >950 (VP1 sequences); at least 14 GI and 17 GII genotypes have been identified (*20*). For SaV, genogroups have only been vaguely defined, mostly because 2 of them (GIV/1 and GV/1) were only recently identified, few sequences exist in the database, and antigenic relationships among all genogroups are unknown. In addition, genetic recombination was only recently discovered and appears to be common within the genus *Sapovirus*.

The purpose of this study was to examine cross-reactivities among the 4 human SaV genogroups and compare results with those of genetic analysis. For this purpose, 2 other SaV strains, GII/3 Syd53 and GIV/1 Syd3, were expressed and antisera were produced against their purified VLPs. A total of 5 SaV strains (GI/1 Mc114, GI/5 Yokote1, GII/3 Syd53, GIV/1 Syd3, and GV/1 NK24) that include all 4 human genogroups and 2 GI genotypes were compared. Our results show that SaV genogroups were antigenically distinct and corresponded with results of genetic classification on the basis of full-length VP1 nucleotide sequences. Proper genetic classification of SaV strains is required, and a consensus of genogroups and genotypes that represent genetically and antigenically diverse strains, which include recombinant SaV strains, should be established to avoid conflicting grouping.

## Materials and Methods

### Specimens

Virus-positive stool specimens were collected from several sources. SaV strain Mc114 (GenBank accession no. AY237422) was isolated from an infant hospitalized with acute gastroenteritis in Chiang Mai, Thailand, in 2001 (*21*). SaV strain NK24 (AY646856) was isolated from an infant with gastroenteritis in Nong Khai, Thailand, in 2003 (*22*). SaV strain Yokote1 was isolated from an outbreak of gastroenteritis at a kindergarten in Yokote City, Japan, in 2006 (*19*). SaV strains Syd53 and Syd3 were isolated from infants hospitalized with acute gastroenteritis in Sydney, New South Wales, Australia, in 2001 (*23*). NoV strain Osaka659 was isolated from an outbreak of gastroenteritis in Japan, in 2006 (unpub. data). RNA extraction and RT-PCR were performed as described (*24*).

### Sequence Analysis

Nucleotide sequences were determined by using the Terminator Cycle Sequence Kit version 3.1 and an ABI 3130 sequencer (both from Applied Biosytems, Boston, MA, USA). Nucleotide sequences were aligned with ClustalX (www.embl.de/~chenna/clustal/darwin) and the distances were calculated by the Kimura 2-parameter method (*24*). Phylogenetic trees with bootstrap analysis from 1,000 replicas were generated by the neighbor-joining method as described (*20*). Amino acid VP1 secondary structure predictions were made by using PSIPRED secondary structural prediction software (*25*).

### Expression of Viruslike Particles

For the expression of VP1 in insect cells, all SaV constructs were designed to begin from the predicted VP1 start AUG codon and included the ORF2 and poly(A) sequences. SaV strains Syd53 and Syd3 were cloned as described (*10*) for strains SaV Mc114, NK24, and Yokote1 according to the protocol of the Baculovirus Expression System using Gateway Technology (Invitrogen, Carlsbad, CA, USA). Briefly, strains Syd53 and Syd3 were amplified with specific sense primers Syd53attb1 (5′-GGGGACAAGTTTGTACAAAAAAGCAGGCTTCGAAGGAGATAGAACCATGGAGGGTGTGTCCCACCCAGA-3′) and Syd3attb1 (5′-GGGGACAAGTTTGTACAAAAAAGCAGGCTTCGAAGGAGATAGAACCATGGAGGGCAATGGCCTACCCCAGGCTG-3′) and antisense primer TX30SXN (5′-GACTAGTTCTAGATCGCGAGCGGCCGCCCTTTTTTTTTTTTTTTTTTTTTTTTTTTTTT-3′). PCR fragments were purified after electrophoresis on a 1.0% agarose gel. Fragments were cloned into donor vector pDONR201 (Invitrogen) and transferred into a baculovirus transfer vector pDEST8 (Invitrogen).

The recombinant pDEST8 was purified and used to transform *Escherichia coli* DH10Bac-competent cells (Invitrogen), which produced recombinant bacmids (baculovirus shuttle vectors) containing the VP1 gene. Recombinant bacmids were then transfected into Sf9 cells (Riken Cell Bank, Ibaraki, Japan), and recombinant baculoviruses were isolated. Recombinant baculoviruses were used to infect ≈3 × 10^6^ confluent Tn5 cells (Invitrogen) at a multiplicity of infection of 5–10 in 1.5 mL of Ex-Cell 405 medium (JRH Biosciences, Lenexa, KS, USA), and the infected cells were incubated at 26°C. The culture medium was harvested 5–6 days postinfection, centrifuged for 10 min at 3,000× *g*, and further centrifuged for 30 min at 10,000× *g*. VLPs were concentrated by ultracentrifugation for 2 h at 45,000 rpm at 4°C (Beckman TLA-55 rotor; Beckman Coulter, Fullerton, CA, USA), and resuspended in 30 μL of Grace’s medium. Samples were examined for VLP formation by electron microscopy as described (*10*), and large-scale production of VLPs was performed as described (*24*).

### Antibody Production

Hyperimmune sera to newly expressed VLPs of SaV (Syd53 and Syd3) were prepared in rabbits and guinea pigs. The first subcutaneous injection was performed with purified VLPs (≈10 μg) in Freund complete adjuvant. After 3 weeks, the animals received 1 booster injection (intravenously in rabbits and subcutaneously in guinea pigs) of 10 μg of VLPs without adjuvant. Blood was collected from the animals 1 week after their last booster injection.

### Antibody ELISA

Cross-reactivities among antiserum samples against SaV were examined by using an antibody ELISA with hyperimmune rabbit antibodies against VLPs. Briefly, wells of 96-well microtiter plates (Maxisorp; Nunc, Roskilde, Denmark) were each coated with 100 μL of purified VLPs (≈1.0 μg/ mL in carbonate-bicarbonate buffer, pH 9.6) (Sigma, St. Louis, MO, USA) and incubated overnight at 4°C. Wells were washed twice with phosphate-buffered saline (PBS) containing 0.1% Tween 20 (PBS-T) and blocked with PBS containing 5% skim milk (PBS-SM) for 1 h at room temperature. Wells were then washed 4 times with PBS-T, 100 μL of 2-fold–diluted hyperimmune rabbit antibodies from an initial concentration of 1:500 in PBS-T-SM was added to each well, and the plates were incubated for 1 h at 37°C. Wells were then washed 4 times with PBS-T, and 100 μL of a 1:1,000 dilution of horseradish peroxidase–conjugated goat antirabbit immunoglobulin G diluted in PBS-T-SM was added to each well. Plates were incubated for 1 h at 37°C. Wells were then washed 4 times with PBS-T, and 100 μL of substrate (*o*-phenylenediamine) and H_2_O_2_ were added to each well, and the plates were left in the dark for 30 min at room temperature. The reaction was stopped by the addition of 50 μL of 2N H_2_SO_4_ to each well, and the absorbance was measured at 492 nm (A_492_). The optical density (OD) cutoff point was determined to be 0.15, which was equal to 3 times the mean OD of preimmune serum (*5*).

### Antigen ELISA

Cross-reactivities among VLPs were also examined by using an antigen ELISA. Briefly, wells were coated with 100 μL of a 1:8,000 dilution of hyperimmune rabbit antiserum diluted in PBS (except for Syd3, for which a 1:3,000 dilution was used), and the plates were incubated overnight at 4°C. Wells were washed 4 times with PBS-T and blocked with PBS-SM for 1 h at room temperature. Wells were then washed 4 times with PBS-T, 100 μL of VLPs (≈1.0 μg/mL in carbonate-bicarbonate buffer, pH 9.6) (Sigma) was added to duplicate hyperimmune rabbit wells, and the plates were incubated for 1 h at 37°C. Wells were then washed 4 times with PBS-T, 100 μL of a 1:8,000 dilution of hyperimmune guinea pig antibody diluted in PBS-T-SM was added to each well (except for Syd3, which used a 1:3,000 dilution), and the plates were incubated for 1 h at 37°C. Wells were washed 4 times with PBS-T, and 100 μL of a 1:1,000 dilution of horseradish peroxidase–conjugated rabbit antiguinea pig immunoglobulin G diluted in PBS-T-SM was added to each well. The plates were then processed as described above. On the basis of our previous study, a specimen with an A_492_ (P – N) >0.1 and a P/N ratio >1.34 (where P is hyperimmune antiserum and N is preimmune antiserum) was considered significantly positive (*4*).

## Results

### Sequence Analysis

**Figure 1:**
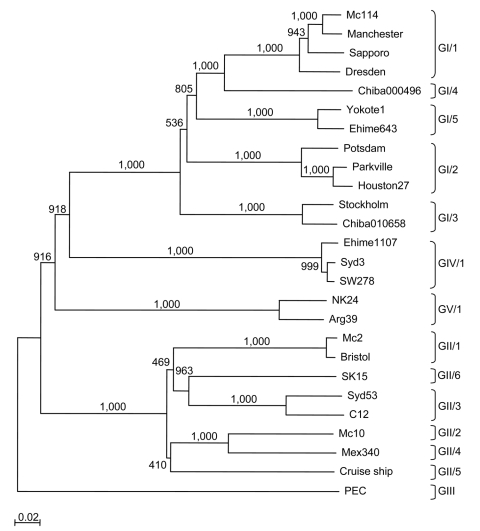
Phylogenetic tree of sapoviruses on the basis of entire nucleotide sequences of the capsid protein. Different genogroups and genotypes are indicated. The numbers on each branch indicate the bootstrap values for the genotype. The scale bar at the lower left represents nucleotide substitutions per site. GenBank accession nos. for reference strains: Arg39, AY289803; Bristol, HCA249939; C12, AY603425; Chiba000496, AJ412800; Chiba010658, AJ606696; Cruise ship, AY289804; Dresden, AY694184; Ehime643, DQ366345; Ehime1107, DQ058829; Houston27, U95644; Manchester, X86560; Mc2, AY237419; Mc10, AY237420; Mc114, AY237422; Mex340, AF435812; NK24, AY646856; Parkville, U73124; PEC, AF182760; Potsdam, AF294739; Sapporo, U65427; SK15, AY646855; Stockholm, AF194182; SW278, DQ125333; Syd3, DQ104357; Syd53, DQ104360; and Yokote1, AB253740.

The sequence of the 3′ end of the genome (≈2,600 nt), i.e., VP1 to poly(A), for the newly expressed SaV strains (Syd53 and Syd3) was determined. Genetic analysis was performed with only complete VP1 sequences, which included sequences from our epidemiologic studies and other sequences available on the database (Figure 1). Five SaV GI and 6 GII genotypes were observed, but only 1 genotype for SaV GIV and 1 for GV was found. This result suggests that SaV GI and GII strains were more genetically diverse, prevalent, or more virulent than SaV GIV and GV strains. However, because the SaV GIV and GV strains were only recently detected (*26*,*27*), this result may reflect only the specificity and sensitivity of the detection methods used. On the basis of our previous classifications, SaV Mc114 and Yokote1 sequences both belonged to GI, but to different genotypes, GI/1 and GI/5, respectively; Syd53 belonged to GII/3; Syd3 belonged to GIV/1; and NK24 belonged to GV/1. SaV GI/1 Mc114 and GI/5 Yokote1 VP1 sequences shared 76.5% and 79% nucleotide similarity and amino acid identity, respectively (Table 1). The nucleotide similarity and amino acid identity among the genogroups was low, i.e., <60% (Table 1).

**Table 1:** Nucleotide similarity (values below blank diagonal) and amino acid identity (values above blank diagonal) of complete capsid (VP1) sequences of sapovirus strains*

Strain	Mc114 (GI/1)	Yokote1 (GI/5)	Syd53 (GII/3)	Syd3 (GIV/1)	NK24 (GV/1)
Mc114 (GI/1)		79	46	52	51
Yokote1 (GI/5)	76.5		46.8	50.3	50.9
Syd53 (GII/3)	56.1	56.9		48.3	48.6
Syd3 (GIV/1)	58.1	57.4	55.9		54.2
NK24 (GV/1)	58.2	57.3	56.4	58.6	

*Values are percentages.

### Expression of VP1

**Figure 2:**
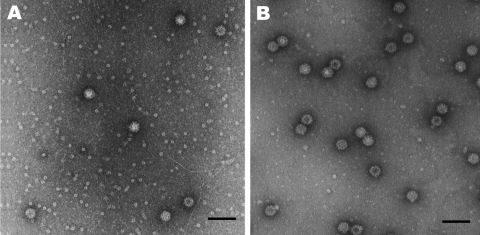
Electron micrographs of A) Syd53 and B) Syd3 viruslike particles of sapovirus. Scale bars = 100 nm.

We previously expressed SaV GI/1 Mc114, GI/5 Yokote1, and GV/1 NK24 in insect cells, which resulted in the formation of VLPs morphologically similar to native SaV (*5*,*10*). In this study, we newly expressed SaV GII/3 Syd53 and GIV/1 Syd3 in insect cells to analyze the cross-reactivity among all human SaV genogroups. SaV GII/3 Syd53 and GIV/1 Syd3 successfully formed VLPs with a diameter of 41 to 46 nm and were morphologically similar to native SaV (Figure 2). Hyperimmune sera against these purified VLPs were prepared in rabbits and guinea pigs.

### Antibody ELISA Analysis

**Figure 3:**
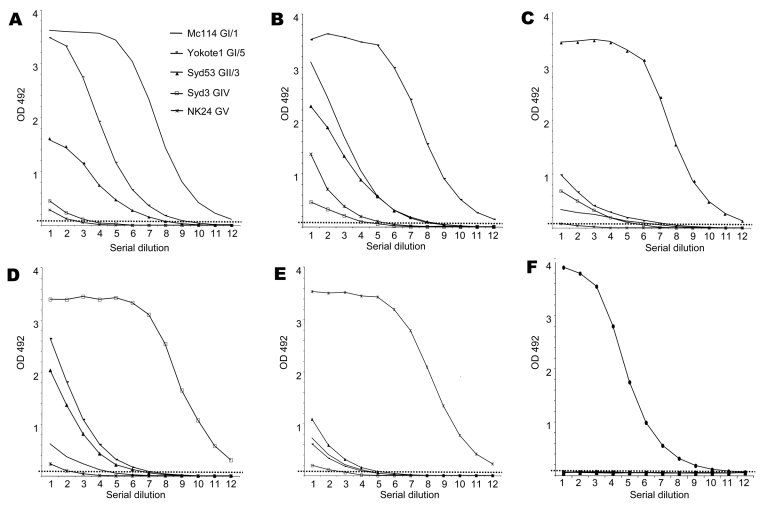
Antibody ELISA absorbances at 492 nm of 6 viruslike particles (VLPs) of sapovirus. A) Mc114 GI/1; B) Yokote1 GI/5; C) Syd53 GII/3; D) Syd3 GIV/1; E) NK24 GV/1; F) NoV Osaka659. Wells were coated with ≈100 ng of purified VLPs. Antiserum was used in 2-fold dilutions from 1:500 to 1,024,000 in phosphate-buffered saline, 0.1% Tween 20, 5% skim milk shown as 1–12 along the x-axis. OD, optical density; NoV, Norwalk virus.

Our previous antibody ELISA result showed that SaV GI/1 Mc114 and GV/1 NK24 antisera had no cross-reactivities against heterologous GV/1 NK24 and GI/1 Mc114 VLPs, respectively (*5*). In the current study, cross-reactivities among 5 VLPs of SaV (GI/1 Mc114, GI/5 Yokote1, GII/3 Syd53, GIV/1 Syd3, and GV/1 NK24) were analyzed by using the antibody ELISA with 2-fold serial dilutions (1:500–1:1,024,000) of hyperimmune antiserum (Figure 3). The OD cutoff point was 0.15, which was equal to 3 times the mean OD of preimmune serum (*5*). Hyperimmune rabbit antiserum reacted strongly against the homologous VLPs (Table 2, Figure 3). SaV GII/3 Syd53 and GIV/1 Syd3 antisera titers were 512,000 and 2,056,000, respectively (Table 2). Several antisera weakly cross-reacted with heterologous VLPs. SaV GI/1 Mc114 antiserum cross-reacted weakly with GI/5 Yokote1 and GII/3 Syd53 VLPs, i.e., their cross-reactivities were 8- and 16-fold lower than that of the homologous VLP titer, respectively. SaV GI/5 Yokote1 antiserum cross-reacted weakly with GI/1 Mc114 and GII/3 Syd53 VLPs, i.e., its cross-reactivity was 16-fold lower than that of the homologous VLP titer. SaV GII/3 Syd53, GIV/1 Syd3, and GV/1 NK24 antisera appeared to have no cross-reactivities against any of the heterologous VLPs, i.e., their cross-reactivities were >32-fold lower than those of the homologous VLP titer. These results suggested that SaV GI/1 Mc114 and GI/5 Yokote1 antiserum had weak 2-way cross-reactivities against GI/5 Yokote1 and GI/1 Mc114 VLPs, respectively. The negative control NoV Osaka659 antiserum showed no cross-reactivities against VLPs of SaV at any dilution of antiserum, which indicates that the antiserum was specific for the VLPs and not the insect cell proteins.

**Table 2:** Titers of antisera to viruslike particles (VLPs) of sapovirus strains in an antibody ELISA

Antisera	VLPs				
	Mc114 (GI/1)	Yokote1 (GI/5)	Syd53 (GII/3)	Syd3 (GIV/1)	NK24 (GV/1)
Mc114 (GI/1)	512,000	64,000	32,000	1,000	<1,000
Yokote1 (GI/5)	32,000	512,000	32,000	2,000	4,000
Syd53 (GII/3)	4,000	8,000	512,000	4,000	<1,000
Syd3 (GIV/1)	2,000	16,000	8,000	2,056,000	1,000
NK24 (GV/1)	2,000	2,000	4,000	1,000	2,056,000

### Antigen ELISA Analysis

On the basis of a previous study, a specimen with an A_492_ (P – N) >0.10 and a P/N ratio >1.34 was considered significantly positive (*4*). Our recent antigen ELISA results showed that SaV GI Mc114, GI/5 Yokote1, and GV/1 NK24 VLPs were antigenically distinct (*19*), i.e., GI/1 Mc114 P– N 0.41, P/N 9.19; GI/5 Yokote1 P – N 0.93, P/N 19.59; and GV/1 NK24 P – N 1.03, P/N 21.58. In the current study, we examined the cross-reactivity among the newly expressed VLPs and those previously prepared by using the antigen ELISA. The antiserum samples reacted only with homologous VLPs, i.e., SaV GII/3 Syd53 P – N 1.02, P/N 21.33; and GIV/1 Syd3 P – N 1.44, P/N 29.74 (Table 3). Several antisera appeared to cross-react with heterologous VLPs, i.e., where the P – N was <0.10, but the P/N ratio was >1.34. SaV GI/5 Yokote1 antisera appeared to cross-react with GII/3 Syd53 VLPs (P/N 1.97); GIV/1 Syd3 antisera appeared to cross-react with GI/5 Yokote1 VLPs (P/N 1.42); and GV/1 NK24 antisera appeared to cross-react with GII/3 Syd53 VLPs (P/N 1.50). However, all of these cross-reactivity results were considered negative because P – N was <0.10.

**Table 3:** Antigen ELISA absorbance values of viruslike particles of sapovirus strains

Antisera	Mc114 (GI/1)	Yokote1 (GI/5)	Syd53 (GII/3)	Syd3 (GIV/1)	NK24 (GV/1)
Mc114 (GI/1)	0.41 (9.19)	0 (1.00)	0 (1.00)	0 (1.00)	0 (1.00)
Yokote1 (GI/5)	0.01 (1.13)	0.93 (19.59)	0.05 (1.97)	0 (1.00)	0 (1.00)
Syd53 (GII/3)	0 (1.00)	0.02 (1.42)	1.02 (21.33)	0.01 (1.14)	0.03 (1.50)
Syd3 (GIV/1)	0 (1.00)	0 (1.00)	0 (1.00)	1.44 (29.74)	0 (1.00)
NK24 (GV/1)	0 (1.00)	0 (1.00)	0 (1.00)	0.01 (1.11)	1.03 (21.58)

*Measured at 492 nm. P, hyperimmune serum; N, preimmune serum.

### Amino Acid Alignment and Secondary Structure Prediction

**Figure 4:**
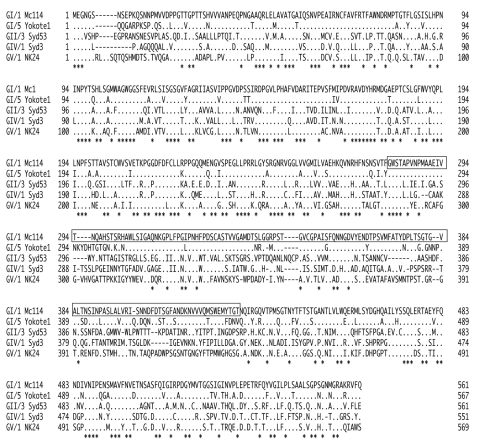
Amino acid alignment of capsid (VP1) sequences of sapoviruses GI/1 Mc114, GI/5 Yokote1, GII/3 Syd53, GIV/1 Syd3, and GV/1 NK24. Sequences in rectangular boxes represent predicted P2 domains (*13*). Asterisks indicate conserved amino acids among these 3 VP1 sequences.

An amino acid alignment of the 5 SaV VP1 sequences (GI/1 Mc114, GI/5 Yokote1, GII/3 Syd53, GIV Syd3, and GV NK24) showed that the shell domain contained more conserved residues than the predicted P domains (Figure 4). However, SaV GI/1 Mc114 and GI/5 Yokote1 shared more conserved continuous residues in the predicted P2 subdomain than other genogroups. The NoV P2 subdomain is thought to contain the determinants of strain specificity, cell binding, and antigenicity. For example, monoclonal antibodies that recognize regions in the P2 subdomain inhibit binding of NoV VLPs to cells (*28*,*29*). In a recent study, we analyzed cross-reactivities among 26 different NoV VLPs (6 GI and 12 GII genotypes) (*30*) and found broad-range cross-reactivities for several NoV antisera. Our results suggested that these cross-reactivities were due to conserved amino acid residues located outside the P2 domain. Conversely, secondary structure predictions made by using PSIPRED secondary structural prediction software showed that helix structures could also influence the cross-reactivity among the NoV VLPs. In the current study, we determined the secondary structure of the 5 SaV VP1 amino acid sequences. Overall, SaV VP1 structures appear to be similar (Appendix Figure). The location of 3 helix structures in the shell domain and 1 helix structure in the C-terminal region were nearly identical for the 5 SaV VP1 sequences. Only SaV GV/1 NK24 was predicted to have a single helix structure in the P2 subdomain. These results suggested that the amino acid sequence, particularly the P2 subdomain, plays a major role in determining cross-reactivity among SaV strains. However, additional studies, including high-resolution VLP structural analysis, are needed.

## Discussion

In this study, we analyzed genetic and antigenic relationships for 4 human SaV genogroups (GI, GII, GIV, and GV). We observed weak 2-way cross-reactivity with SaV GI/1 Mc114 and GI/5 Yokote1 antisera against the heterologous GI/5 Yokote1 and GI/1 Mc114 VLPs, respectively, by using an antibody ELISA. However, when we used an antigen ELISA, we found that GI/1 Mc114 and GI/5 Yokote1 VLPs were antigenically distinct. These weak cross-reactivities identified by using the antibody ELISA may have been influenced by several factors, including unfolded VLPs on the microtiter plates at the high pH (carbonate-bicarbonate buffer, pH 9.6) (*31*) or conserved continuous residues outside the predicted P2 domain. Therefore, these 2 SaV genotypes (GI/1 and GI/5) are for the most part antigenically distinct. Likewise, we found that the 4 human SaV genogroups were antigenically distinct in the antigen ELISA. To our knowledge, these new findings provide the first evidence that SaV antigenicity corresponded well with VP1 genogrouping and genotyping.

## Supplementary Material

Appendix Figure Schematic representations of complete predicted secondary structure of sapoviruses GI/1 Mc114, GI/5 Yokote1, GII/3 Syd53, GIV/1 Syd3, and GV/1 NK24 VP1. The first line shows level of confidence of prediction (Conf), where 10 represents high and 0 represents low confidence of prediction. The second line shows predicted secondary structure (Pred), where helix is indicated by a green cylinder, β strand by a yellow arrow, and coil by a line. The third line also shows predicted secondary structure (Pred), where helix is indicated by an H, β strand by an E, coil by a C. The fourth line shows amino acid (AA) sequence.
