# Supplementary figures and images for: Antigenic Diversity of Human Sapoviruses
Source: Emerg Infect Dis. 2007 Oct;13(10):1519–25. doi: 10.3201/eid1310.070402 (PMC2851512; doi:10.3201/eid1310.070402)

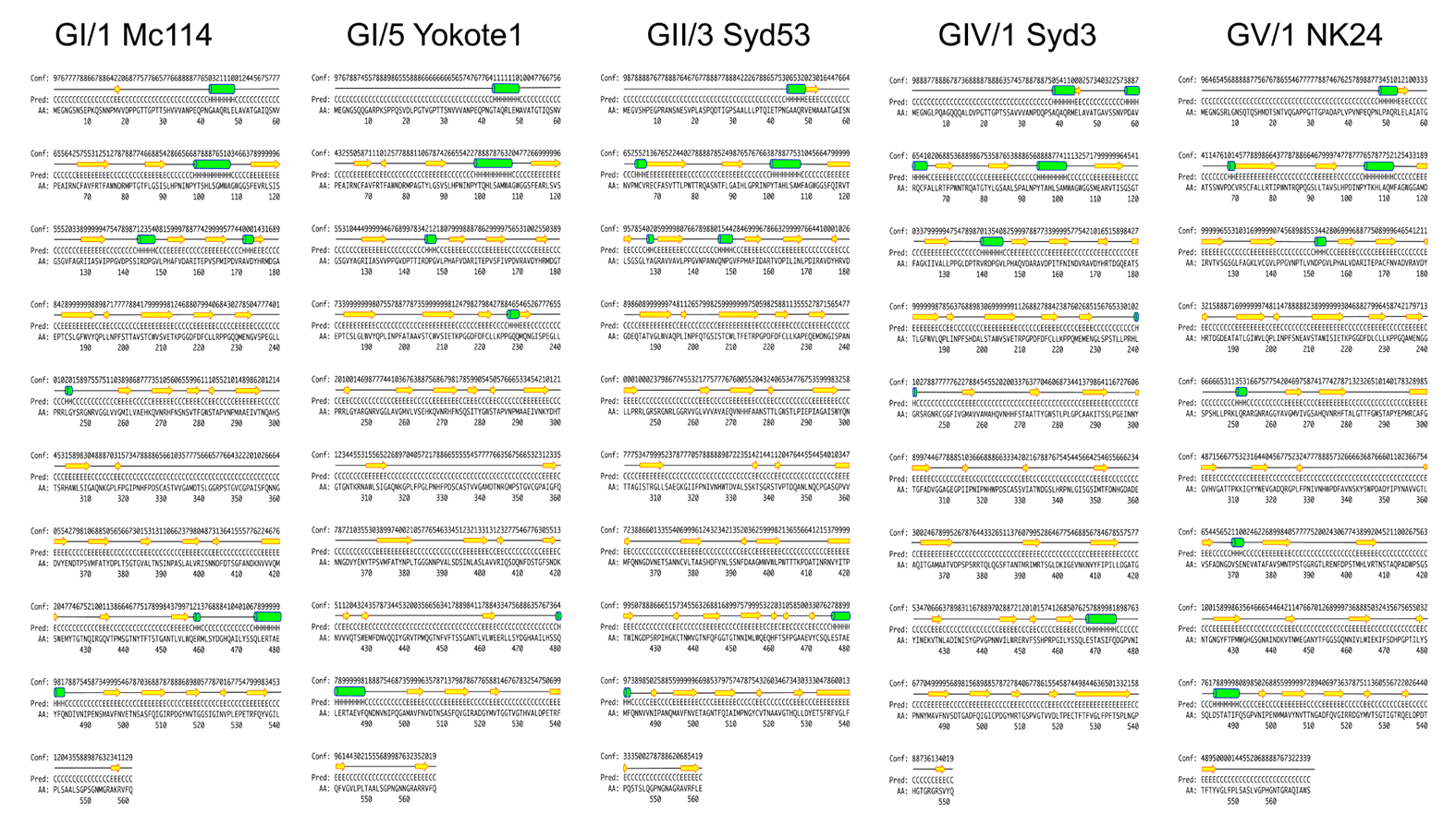

Supplement: Appendix Figure — Schematic representations of complete predicted secondary structure of sapoviruses GI/1 Mc114, GI/5 Yokote1, GII/3 Syd53, GIV/1 Syd3, and GV/1 NK24 VP1. The first line shows level of confidence of prediction (Conf), where 10 represents high and 0 represents low confidence of prediction. The second line shows predicted secondary structure (Pred), where helix is indicated by a green cylinder, β strand by a yellow arrow, and coil by a line. The third line also shows predicted secondary structure (Pred), where helix is indicated by an H, β strand by an E, coil by a C. The fourth line shows amino acid (AA) sequence. [file 07-0402_appF-s1.gif]
